# Statin-Associated Headache: A Rare and Underrecognized Clinical Presentation: A Case Report

**DOI:** 10.3390/reports9010007

**Published:** 2025-12-24

**Authors:** Mohammad. I. Ullah, Sadeka Tamanna

**Affiliations:** 1 University of Mississippi Medical Center, 2500 N State St., Jackson, MS 39216, USA; 2 G. V. (Sonny) Montgomery VA Medical Center, 1500 E Woodrow Wilson Ave., Jackson, MS 39216, USA; sadeka.tamanna@va.gov

**Keywords:** Headache, Statin, Simvastatin

## Abstract

**Background** **and Clinical Significance**: Statins are widely prescribed for cardiovascular risk reduction and generally demonstrate a favorable safety profile. While myalgia and elevations in liver enzymes are well-recognized adverse effects, headaches are less commonly reported and often underrecognized in clinical practice. This may result in unnecessary diagnostic evaluations, increased healthcare costs, and delayed identification of the underlying cause. **Case Presentation**: We describe an adult patient who developed intractable headaches that emerged after many years of statin therapy. The headaches persisted despite conventional analgesic treatment and resolved completely following discontinuation of the statin. Secondary causes were excluded, and comorbid conditions were systematically ruled out. Statin-associated headache is uncommon but clinically relevant. Proposed mechanisms include nitric-oxide-mediated vasodilation, central effects of lipophilic statins, and mitochondrial involvement. In this case, the patient was taking metoprolol succinate, lisinopril, simvastatin, clopidogrel, and tamsulosin. Except for lisinopril, none of the other comedications are strongly linked to new-onset headaches. Holding it did not resolve his headache, making simvastatin the most plausible contributor. This was confirmed by resolution of headache through its discontinuation. Because such headaches may be overlooked, clinicians should consider a statin-related cause when symptoms begin after initiation and may manage this by switching to a hydrophilic statin or using alternative lipid-lowering therapy. **Conclusions:** Clinicians should remain vigilant about the possibility of statin-induced headache, even in long-term users. Early recognition can prevent unnecessary diagnostic investigations, expedite symptom resolution, and support optimal management of both cardiovascular risk and treatment-related adverse effects.

## 1. Introduction and Clinical Significance

Statins are widely prescribed as first-line agents for the management of hyperlipidemia and the prevention of cardiovascular disease. Their safety profile is well-established, with adverse effects most frequently involving the musculoskeletal and hepatic systems. Neurological side effects, including headaches, are less well-characterized and are not typically emphasized in the literature. There is no high-quality evidence that establishes a clear “proportion of headaches” caused by statins overall, because headaches are common in the general population and many reported events in patients on statins occur just as often on placebo. However, a few trial and meta-analytic data provide some rough estimates. Randomized controlled trials consistently show that headache occurs in approximately 5–6% of statin-treated patients, a rate nearly identical to placebo groups (~5%), with absolute risk differences typically 0–1%. This corresponds to an estimated 5–11 potentially statin-attributable headaches per 1000 patients, indicating that while possible, headache is an uncommon and often difficult-to-attribute adverse effect [1,2].

Although most statin-associated adverse effects appear shortly after initiation, post-marketing pharmacovigilance data and case reports show that some adverse events may have a variable time to onset, sometimes occurring weeks to months after starting therapy or during long-term use; however, such observations come primarily from spontaneous reports and should be interpreted with caution [3,4,5]. In such instances, establishing a potential causal link requires careful consideration, as both clinicians and patients may initially overlook the association.

We describe a case of statin-associated headache that developed after years of uneventful therapy and resolved upon drug withdrawal, highlighting a rarely reported but clinically relevant phenomenon. The aim of this report is to raise awareness among clinicians regarding the possibility of delayed statin-associated headaches and to contribute to the limited literature on the neurological side effect profile of statins.

## 2. Case Presentation

A 64-year-old Caucasian male patient came to see his primary care provider (PCP) for his 6-month follow-up visit. He had a history of hypertension, hyperlipidemia, and coronary artery disease (status post-stent placement 5 years back). He also had benign prostatic hyperplasia (BPH) with increased urinary frequency but no history of urinary retention. He stated that he had been having intermittent headaches for the last 3–4 months. He had taken acetaminophen as needed, and it used to work for him initially. But, for the last month, the headaches had been dull but constant, and the acetaminophen was not helping. The pain was mostly in the frontal region, but it often involved the occipital region, as well. He had no prior history of migraine. The pain was not associated with photophobia, nausea, or vomiting. It was not aggravated by change in posture or neck movement. The pain improved on its own some days, but it kept coming back and stayed as a dull, nagging pain. It often increased without any specific factor that he could identify. He did not smoke or drink alcohol. He drank one cup of coffee in the morning only. He skipped coffee for a few weeks to see if his headache improved, but it did not make a difference. He did not use any artificial sweetener in his coffee. He did not eat cheese or use monosodium glutamate (MSG) in his food.

His current medications included metoprolol succinate 50 mg daily, lisinopril 10 mg daily, simvastatin 20 mg daily, clopidogrel 75 mg daily, and tamsulosin 0.4 mg daily. His medication regimen and dosage had not changed in the last 2 years. He was not taking any over the counter medication or supplement. His blood pressure was well-controlled and ran mostly <125/80 mm Hg at home and at the doctor’s office. His physical examinations, including cardiovascular and neurological examinations, were normal. He had no pain or tenderness in his neck movements in any direction. He had seen his eye doctor 3 months back, and his eyeglasses prescription had been updated.

His routine blood tests, including CBC, complete metabolic panel, ESR, TSH, HbA1c, and urine toxicology, were all within normal limits. His polypharmacy, especially the use of lisinopril and tamsulosin together, sometimes caused drops in blood pressure and headache or dizziness. Lisinopril itself may sometimes cause headache. Therefore, he was advised to stop taking Lisinopril for the next 2 weeks to see if it was causing the headache. He called back after 2 weeks stating that his headache had not changed at all. He was advised to restart back on Lisinopril, and an MRI of the head was ordered. It did not show any space-occupying lesion or other abnormal finding that might explain his headache. He was referred to neurology for further evaluation.

In neurology, he had an MR angiography to rule out vascular causes (such as aneurysm or venous sinus thrombosis), and it was normal. A lumbar puncture was performed that showed normal opening pressure and normal CSF findings. An EEG was performed to rule out atypical seizure, and it was normal. A polysomnography was performed, as he had a history of intermittent mild snoring. His apnea hypopnea index (AHI) was only 1.2/h, and obstructive sleep apnea was ruled out.

According to the International Classification of Headache Disorders, 3rd edition (ICHD-3), and supported by an unrevealing secondary headache workup, the patient’s presentation was most consistent with chronic tension-type headache (CTTH). His headaches were bilateral, pressing, non-pulsating, and of mild to moderate intensity, without photophobia, phonophobia, nausea, vomiting, or neurological deficits, and were not worsened by routine physical activity. The near-daily pattern for over three months meets the chronicity criterion for CTTH. These features, particularly the non-pulsatile quality, absence of sensory sensitivities or gastrointestinal symptoms, and lack of activity-related aggravation, make the presentation incompatible with migraine without aura or other primary headache disorders under ICHD-3 definitions.

He was started on amitriptyline 10 mg nightly, and it was titrated up to 50 mg over the next 3 months. He was closely monitored for side effects due to his history of BPH. He could not tolerate any higher dose due to having dry mouth and feeling groggy during the day. He continued to take amitriptyline 50 mg nightly but still had dull headaches on most days, even though the intensity was less severe than before. He came back to his primary care doctor and expressed his frustration with his headache. He was reassured after seeing the neurologist that he did not have any life-threatening disease, but he was depressed because of his ongoing headache. He requested to do something different so that he could get some relief. 

**Figure 1:**
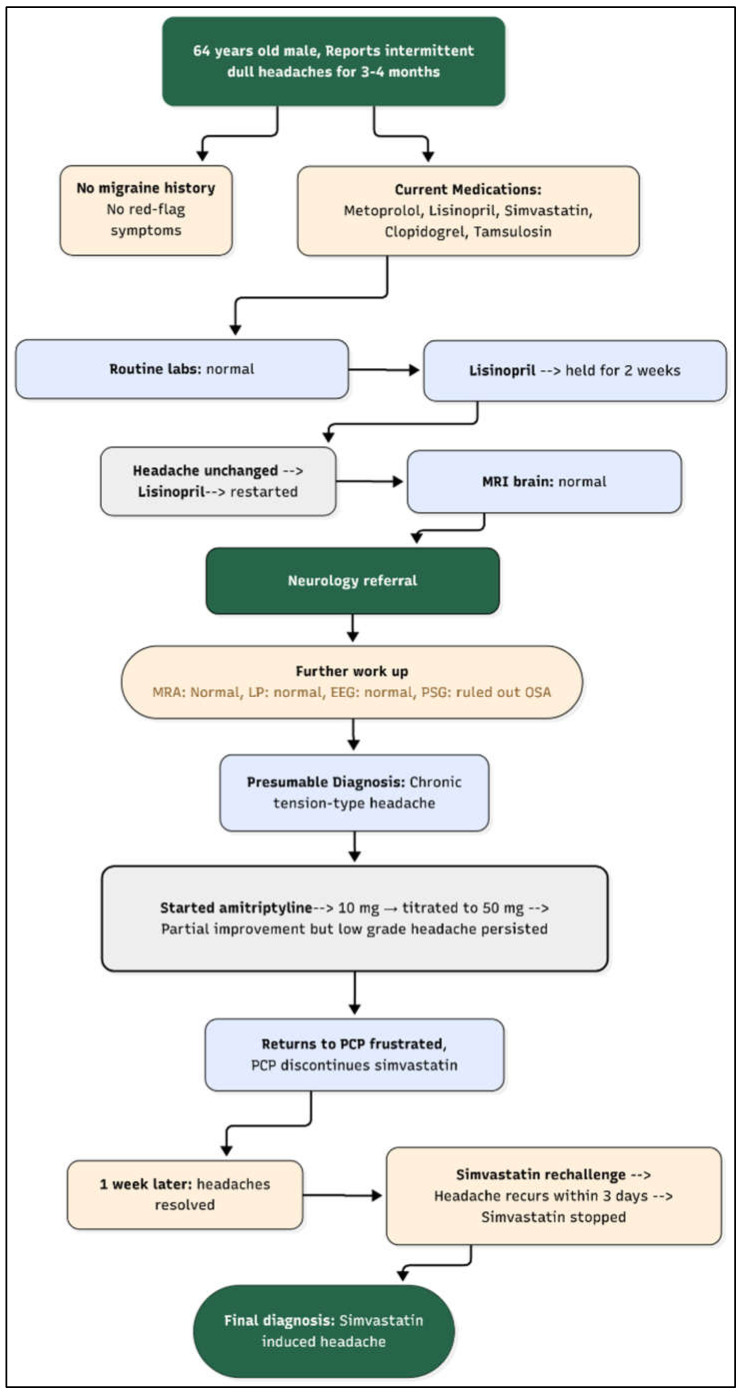
Flow diagram of clinical timeline. **Legend:** OSA: obstructive sleep apnea, MRA: magnetic resonance angiography, MRI: magnetic resonance imaging, LP: lumbar puncture, EEG: electroencephalography.

After reviewing his medical history, his recent thorough lab investigations, and imaging, his PCP decided to hold each of his current medications systematically, one by one, to rule out possibility of medication-induced headaches. Headache has been listed as potential side effect of statin and his other medications as well, but it is not commonly seen in clinical practice. Beta blockers and tricyclic antidepressants may also modulate the headache phenotype. The PCP decided to hold the statin first (simvastatin) to see if it was contributing to his headache. He called back after one week stating that he no longer had headaches. He was then advised to restart simvastatin to see if his headache recurs. Within 3 days, his headache came back, and the quality was very similar to the headache he had been having before he stopped his simvastatin. He stopped the medication on his own. He was very pleased that he was now headache-free after such a long time and was not interested in trying any alternative statin therapy. On his follow-up visit after 3 months, he was again encouraged to try an alternative statin due to his history coronary artery disease, and he agreed to try Rosuvastatin. He was started on it at 10 mg daily and did not experience headache anymore. After one year, his lipid panel stayed at goal for his coronary artery disease on this regimen. He did not experience headaches or any other side effects from it. Figure 1 shows the timeline of events of this clinical case.

## 3. Discussion

Headache is among the most common neurological complaints and classified into primary and secondary headache disorders. In our patient, the differential diagnoses included primary causes, most notably chronic tension-type headache and migraine, as well as a targeted set of secondary etiologies, such as medication-related headache, intracranial structural causes, vascular disorders, CSF pressure abnormalities, and sleep-related conditions. These possibilities are summarized in Table 1, which outlines the diagnostic considerations and rationale for excluding each condition based on clinical features and the results of a comprehensive workup.

Because medication effects are a frequent and sometimes underrecognized cause of persistent headache, we also reviewed the patient’s chronic medications. Table 2 focuses specifically on drug classes relevant to this case, statins, antihypertensives, antiplatelet agents, tamsulosin, and amitriptyline, and highlights their known associations with headaches.

Early-onset headaches typically arise acutely and are most often related to primary headache disorders, such as migraine or tension-type headache, though they may also reflect acute vascular or inflammatory processes. Proposed mechanisms include nitric-oxide-mediated vasodilation, CGRP-driven trigeminovascular activation, and transient meningeal or intracranial pressure changes [6,7]. Migraine is relatively common, affecting approximately 8–12% of the population, while truly sudden (“thunderclap”) headaches remain rare at about 43 per 100,000 person-years [7].

Late-onset headaches, particularly those developed after age 50, raise greater concern for secondary causes. They may result from vascular disease (e.g., giant cell arteritis), neoplastic processes, structural brain changes, altered cerebrospinal fluid dynamics, or medication overuse, with mechanisms involving neuroinflammation, meningeal stretching, or central sensitization [8,9]. New headaches occur in up to 10% of adults over 55, with giant cell arteritis accounting for 18–28 cases per 100,000 individuals annually in this age group [8]. Because of the greater likelihood of secondary pathology, late-onset headaches are considered “red flags” requiring targeted evaluation [10].

Statins are among the most widely prescribed medications worldwide for the treatment of hyperlipidemia and cardiovascular risk reduction. Their safety profile is generally favorable, with adverse events being relatively uncommon and often manageable. The most frequently reported side effects include myalgia, myopathy, hepatotoxicity, gastrointestinal disturbances, and, less commonly, cognitive changes [11,12]. Neurological side effects, such as peripheral neuropathy, dizziness, insomnia, and neurocognitive complaints, have been described with statin use [11,13]. Although headaches are occasionally documented in clinical trials, it is not typically regarded as a prominent or serious statin-associated adverse effect.

**Figure 2:**
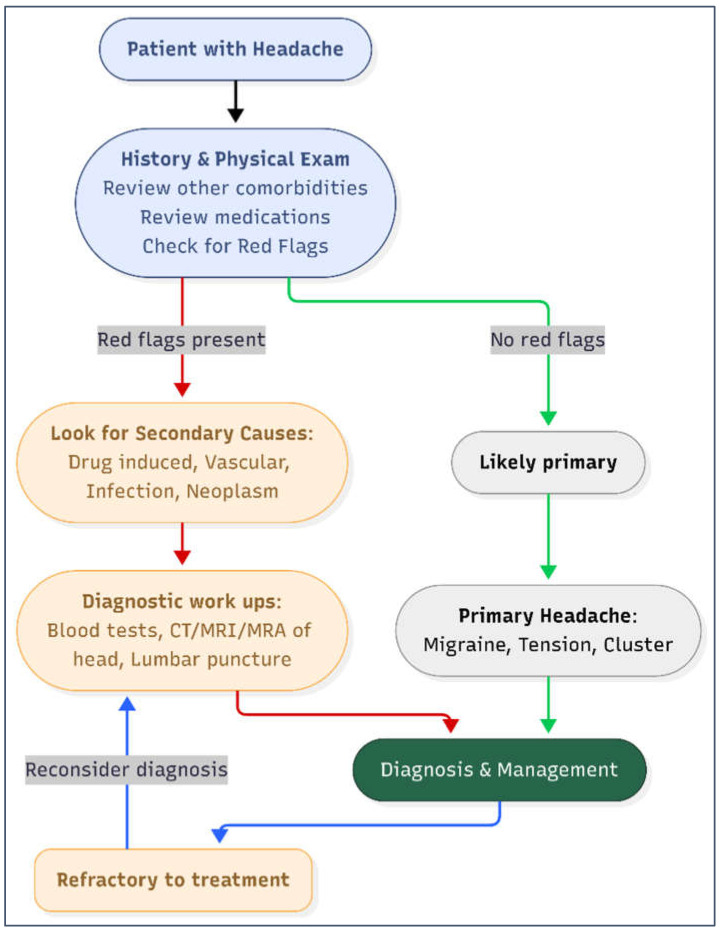
Diagnostic work-up of headache in adults. Diagnostic red flags include systemic symptoms, neurological deficit, sudden onset, older age, progressive or positional in nature, papilledema. **Legend:** MRA: magnetic resonance angiography. MRI: magnetic resonance imaging.

The diagnostic approach for headache should begin with a careful history and neurological examination, with particular attention to temporal associations between symptom onset and medication use (Figure 2). Laboratory tests (e.g., CBC, ESR/CRP, thyroid function, vitamin levels) and neuroimaging (CT or MRI) are reserved for patients with “red flags” or suspected secondary causes, while additional evaluations, such as lumbar puncture or vascular imaging, are guided by clinical suspicion [14].

A systematic, stepwise evaluation helps distinguish benign primary headaches from secondary or drug-related causes that require modification or discontinuation of therapy [10]. In our case, a stepwise diagnostic approach was used consistent with recommendations from the U.S. Headache Consortium, the American Academy of Neurology (AAN), and published reviews on evidence-based headache evaluation. Neuroimaging with MRI was obtained because the patient was over 50 years old with persistent chronic headaches and required exclusion of secondary causes; this is recommended in cases with non-acute but unexplained headache presentations. MRA was added to assess vascular etiologies, including aneurysm or dissection, particularly because vascular causes may present with persistent headache despite unremarkable MRI findings.

Because symptoms persisted despite normal neuroimaging, a lumbar puncture was performed to evaluate for presence of infection, inflammation, abnormal CSF pressure, and other secondary conditions. EEG was obtained to rule out atypical seizure activity, consistent with AAN guidance that EEG may be appropriate when episodic neurological symptoms or diagnostic uncertainty remains. Polysomnography was pursued because obstructive sleep apnea is a recognized contributor to chronic daily headaches and the patient reported intermittent snoring. This diagnostic cascade reflects a structured, guideline-based evaluation to exclude secondary headache causes before diagnosing chronic tension-type headache [15,16,17,18,19,20].

### 3.1. Incidence of Headache with Statins

Clinical trial and post-marketing surveillance data suggest that headaches occur in a minority of statin users, though exact prevalence rates vary across agents. Headache incidence is generally reported at 2–7% depending on the statin and study population [12,21]. For instance, simvastatin has reported rates of headache around 3–5%, atorvastatin 2–4%, and rosuvastatin 2–3% [22,23]. Compared to more common adverse effects, such as muscle-related symptoms, headache remains an underappreciated clinical finding.

Published pharmacovigilance analyses show that headache is among the nervous system events reported with statin therapy [24]. Some statin-associated adverse events reported in pharmacovigilance databases demonstrate onset ranging from days to many months after initiation; however, robust evidence specifically supporting ‘years-after’ onset of statin-related headache is limited, and such delayed presentations largely reflect observational reports rather than controlled studies [4,25,26]. However, peer-reviewed case reports that document a clear withdrawal–resolution–rechallenge sequence for headache specifically are scarce. The strongest clinical evidence for statin causality generally comes from dechallenge–rechallenge observations in other statin adverse events (e.g., myopathy, cognitive/psychiatric complaints), and this same approach was applied in our case to support a probable relationship.

From a management standpoint, this case illustrates that suspected statin intolerance does not necessarily preclude future statin therapy. Although the patient initially declined alternative agents after symptom resolution, he later agreed to trial rosuvastatin, which he tolerated without recurrence of headache while maintaining lipid control. This highlights the importance of patient counseling and consideration of alternative statins when adverse effects are suspected.

### 3.2. Potential Mechanisms

The pathophysiological mechanisms of statin-induced headache are not well-defined, but several hypotheses exist. Proposed mechanisms include upregulation of endothelial nitric oxide synthase (eNOS), which increases nitric oxide availability and promotes cerebral vasodilation [27], enhanced blood–brain barrier penetration by lipophilic statins, such as simvastatin and atorvastatin [28], and subtle mitochondrial alterations or coenzyme Q10 depletion [29]. Susceptibility may be influenced by genetic variants, including SLCO1B1 polymorphisms that alter statin pharmacokinetics [30] and NOS3 variants that modulate vascular reactivity [31]. Demographic factors may also contribute; women and younger adults tend to experience medication-related headaches more frequently due to hormonal and vascular factors [32], while racial differences in CYP3A4 and CYP3A5 metabolism may underlie variable adverse effect profiles [33].

It is important to note that the mechanistic explanations for statin-associated headache described above are theoretical and not specifically demonstrated in this patient. We did not perform genetic testing or biochemical assays of mitochondrial or endothelial function, and therefore no definitive susceptibility factor can be established. These mechanisms are presented to contextualize potential pathways proposed in the literature, but the patient’s clinical course, particularly the reproducible withdrawal–rechallenge pattern, remains the primary basis for attributing his headache to statin therapy.

### 3.3. Comparison with Other Headache-Inducing Medications

Placing statins in the broader context of drug-induced headaches highlights important clinical differences. Medications such as nitrates and phosphodiesterase-5 inhibitors are well-known to cause headaches in up to 30–50% of users due to nitric-oxide-mediated vasodilation [34,35,36]. Similarly, calcium channel blockers cause vasodilatory headaches in 5–10% of patients [37], while SSRIs and oral contraceptives contribute to headaches in 10–25% of users through serotonergic and hormonal mechanisms [38,39,40]. Some agents, such as topiramate, may paradoxically both induce and prevent headaches depending on dose and patient characteristics [41]. Unlike these drugs, statins lack a primary vasodilatory or neurotransmitter-modulating action that would predictably induce headaches. This makes statin-related headaches a more idiosyncratic and patient-specific phenomenon, likely mediated by individual genetic and metabolic factors rather than a universal drug property.

## 4. Conclusions

This case highlights the importance of having awareness regarding the potential for new onset of statin-induced headache, even after long-term therapy. Clinicians should have a high index of suspicion when evaluating persistent headaches in patients receiving statins, particularly after excluding other common causes. Timely recognition of this adverse effect can minimize unnecessary diagnostic testing and allow for faster symptom resolution. For patients with significant or refractory headaches, switching to a hydrophilic statin (e.g., pravastatin, rosuvastatin) or considering alternative lipid-lowering therapies may improve tolerability while maintaining cardiovascular protection.

### Key Learning Points

• Headache is an uncommon but possible adverse effect of statins, including simvastatin.
• Potential mechanisms include nitric-oxide-mediated vasodilation, lipophilic CNS penetration, and mitochondrial effects.
• Clinical management may include switching to hydrophilic statins or alternative therapies.

## Figures and Tables

**Table 1:** Focused differential diagnosis considered in this patient with chronic daily headache, with reasons for exclusion.

Category	Diagnosis	Key Clinical Features	Reason for Inclusion or Exclusion
**Primary Headache Disorders**	Chronic tension-type headache (CTTH)	Bilateral, pressing, non-pulsating, mild–moderate, not worsened by activity; no photophobia, phonophobia, nausea, or aura	Most consistent with patient’s phenotype. Headache present >3 months, normal neurologic exam, meets ICHD-3 criteria.
	Migraine without aura	Unilateral, pulsatile, moderate–severe; nausea, photophobia, or phonophobia common	Excluded: lacks pulsatile quality, sensory sensitivities, and GI symptoms.
	Cervicogenic headache	Neck-related pain, worsens with neck movement; cervical tenderness or restricted ROM	Excluded: normal cervical exam; no positional component.
**Secondary Headache Disorders**	Medication-related headache (including statins)	Headache temporally related to medication initiation or discontinuation	Most clinically relevant: headache resolved after stopping simvastatin and recurred with rechallenge. Strong causal relationship.
	Medication-overuse headache	Near-daily headache with analgesic use >10–15 days/month	Excluded: only intermittent acetaminophen use.
	Intracranial structural causes (mass lesion, Chiari, hydrocephalus)	Progressive headache, neurologic deficits, morning predominance	Excluded: MRI of brain normal; exam normal.
	CSF pressure disorders (IIH, low-pressure headache)	Positional headache, visual symptoms, papilledema	Excluded: normal LP opening pressure; no visual symptoms.
	Vascular causes (aneurysm, dissection, venous sinus thrombosis)	Thunderclap onset, focal deficits, persistent atypical headache	Excluded: MRA head/neck normal; no thunderclap pattern.
	Sleep-apnea–related headache	Morning headache, snoring, daytime sleepiness	Excluded: polysomnography AHI 1.2; OSA ruled out.

**Table 2:** Medications associated with headache (pertinent to this patient).

Medication Class	Examples	Headache Frequency	Relevance to This Case
**Statins**	Simvastatin, atorvastatin	2–6% in RCTs; excess risk ~0.5–1% above placebo	Most relevant: withdrawal–rechallenge confirmed causality.
**ACE inhibitors**	Lisinopril	1–5%	Considered due to polypharmacy; temporary discontinuation did not reduce headache.
**Beta blockers**	Metoprolol	<1%	Not typically headache-inducing; continued without issues.
**Alpha-1 antagonists**	Tamsulosin	1–2%	May cause dizziness or BP changes; no temporal relationship.
**Antiplatelet agents**	Clopidogrel	Rare (<1%)	Not typically associated with chronic daily headache.
**Tricyclic antidepressants**	Amitriptyline	Can cause worsening headache initially but used for prophylaxis	Ineffective at adequate doses; limited by side effects.

## Data Availability

The original contributions presented in this study are included in the article. Further inquiries can be directed to the corresponding author.
